# PFAS Modulate Osmotic Signaling Independent of Gravimetric Changes in the Rat Uterus

**DOI:** 10.3390/toxics12030170

**Published:** 2024-02-23

**Authors:** Aaron Dixon, Evelyn G. Rowan, Allison N. Yackley, Erin P. Hines

**Affiliations:** 1 Reproductive and Developmental Toxicology Branch, Public Health Integrated Toxicology Division, Center for Public Health and Environmental Assessment, Office of Research and Development, United States Environmental Protection Agency, Research Triangle Park, NC 27711, USA; dixon.aaron@epa.gov; 2 Oak Ridge Institute for Science and Education, U.S. Department of Energy, Oak Ridge, TN 37831, USA; rowan.evelyn@epa.gov (E.G.R.); a_yackley@yahoo.com (A.N.Y.)

**Keywords:** PFAS, uterus, reproductive toxicology, endocrine disruptors, cancer signaling

## Abstract

Various PFAS have been identified as potential endocrine-disrupting chemicals due to estrogen receptor activation, impacts on puberty timing, or impacts on hormonally sensitive endpoints in fish. This study screened multiple PFAS in the rat uterotrophic assay to determine potential estrogenic effects on the uterus with PFAS exposure. This study also explored PFAS-dependent uterine signaling with an osmotic stress mRNA gene expression array. Briefly, Sprague–Dawley rats (26–39 days old) were ovariectomized, and uterine tissue was allowed to regress for a 3-week period of recovery. Animals were then exposed daily via oral gavage to PFAS for 4 days, and then uterine weight was determined. In contrast to the positive control estrogens, the PFAS tested (4:2, 6:2, and 8:2FTOH; perfluorooctanesulfonamide (PFOSA), perfluorononanoic acid (PFNA), perfluorohexane sulfonate (PFHxS), perfluorooctane sulfonate (PFOS), nafion byproduct 2 (NBP2), 1H,1H,8H,8H-perfluorooctane-1,8-diol (FC8-diol) and 1H,1H,10H,10H-perfluorodecane-1,10-diol (FC10-diol)) caused no significant changes in the uterine weight. Hormonally active compounds can act as carcinogens, and because earlier rodent work has demonstrated that chronic PFOA exposure is associated with increased risk of uterine cancer, uterine mRNA gene expression was explored with an osmotic stress RT-qPCR array. PFAS exposure significantly upregulated multiple genes across the array, with PFOSA being the compound most similar to the reference estrogens (estradiol benzoate and ethinyl estradiol) in its expression pattern. Also, across all PFAS, pathway analysis revealed that the paxillin pathway, a pathway important in tumor suppressor gene SHP-2 signaling, was significantly upregulated with PFAS exposure. These results demonstrate that in vitro estrogen screens or impacts in fish may show different responses from direct impacts on mammalian uterine weight as assessed with the uterotrophic assay. This study also builds out new mechanisms that may contribute to understanding of carcinogenic changes seen in the uterus after PFAS exposure.

## 1. Introduction

Per- and polyfluoroalkyl substances (PFAS) are a large class of fluorinated organic chemicals that are ubiquitous and persistent in humans and the environment and are thus of interest to regulatory agencies and the general public. Multiple PFAS have been identified as potential endocrine-disrupting chemicals [1] due to estrogen receptor activation [2,3,4], impacts on puberty timing [5,6,7,8]. In this study, the uterotrophic assay was used to address the impact of PFAS as a mammalian endocrine disruptor. The uterotrophic assay is one of hundreds of OECD guideline tests that have been developed to test the potential hazard of chemicals. These guideline tests are internationally recognized and used by government, industry, and independent labs to identify potentially hazardous chemicals. The OECD guideline 440 uterotrophic assay is also used by the US EPA in its Endocrine Disruptor Screening Program. The uterotrophic assay and other guideline tests are used within regulatory science as steps needed for chemical product notification and registration. Previous work in other labs identified PFAS with estrogenic activation in fish or estrogen receptor activation with in vitro screens. Villeneuve et al. [7] used tiered testing, starting with in vitro estrogen receptor screening and further testing in fish for the estrogen sensitive endpoint of vitellogenin activation. Of the five PFAS (perfluorooctanoic acid [PFOA]; 1H,1H,8H,8H-perfluorooctane-1,8-diol [FC8-diol]; 1H,1H,10H,10H-perfluorodecane-1,10-diol [FC10-diol]; 1H,1H,8H,8H-perfluoro-3,6-dioxaoctane-1,8-diol [FC8-DOD]; and perfluoro-2-methyl-3-oxahexanoic acid [HFPO-DA]) that were positive estrogen receptor activators in vitro, only three induced vitellogenin activation (FC8-diol, FC10-diol, and FC8-DOD) [7]. Evans et al. [2] reported in vitro human estrogen receptor activation with four PFAS (PFHxS, 8:2 FTOH, 6:2 FTOH, and PFOSA) from the sixteen PFAS tested. To build upon this earlier non-mammalian work, the current study evaluated the potential estrogenicity of PFAS with the rat uterotrophic assay in which animals had been ovariectomized and did not produce endogenous estrogens, leaving the regressed uterus sensitive to administered hormonal substances. The uterus can respond to hormonal activation in two ways, with increased fluid or water uptake and with increased tissue growth. This work was conducted to assess the potential in vivo estrogenic activity of various PFAS in a mammalian model. Some of the PFAS tested herein induced transcriptional activation of estrogen receptor with the in vitro assays [2] or induced vitellogenin activation in fish [7,9]. Tiered testing beginning with in vitro screening and building out to the in vivo uterotrophic model allows for a multiple-level approach to investigate the hormonal activity of these PFAS. The uterotrophic assay was used to screen for in vivo estrogenic effects in a mammalian species after PFAS exposure to compounds that demonstrated ER activity in vitro.

PFAS-dependent changes to the uterus may manifest with or without direct impact on uterine weight. Uterine cancer has been reported in rodents with chronic exposure to PFAS in the two-year cancer bioassay [10]. To expand the understanding of potential PFAS effects on the uterine signaling, PFAS-mediated gene expression changes were measured with a RT-qPCR array that measured genes involved in osmotic signaling after an acute (4-day) exposure. PFAS have been associated with multiple uterine pathologies in human epidemiologic studies, including endometriosis [11,12], endometriosis-related infertility [11], menstrual irregularities [13], and altered age at menarche [14,15]. In addition to the gravimetric or weight changes measured with the uterotrophic assay, an osmotic gene array and pathway analysis was used to build out potential adverse outcome pathways in the uterus after PFAS exposure.

The International Agency for Research on Cancer (IARC), the cancer agency of the World Health Organization (WHO), has evaluated the carcinogenicity of perfluorooctanoic acid (PFOA) and perfluorooctanesulfonic acid (PFOS) and classified them as carcinogens (Class 1 and Class 2, respectively) [16]. PFOA and PFOS are the two most extensively studied PFAS, but there is now recognition that tens of thousands of PFAS exist in the environment and commerce. The PFAS PFOA and PFOS are also listed as persistent organic pollutants and regulated as such by the Stockholm Convention [17]. The NTP previously conducted a two-year cancer bioassay with chronic exposure of rodents to PFOA [10]. In the NTP study, the incidence of uterine adenocarcinoma was significantly elevated with PFOA exposure. Because PFAS like PFOA are neither mutagenic nor genotoxic in nature [10,18,19] exploring other potential carcinogenic signaling pathways is important to understand this increased incidence of uterine cancer reported in earlier studies. Previous work in cell culture models showed SHP-2 and paxillin pathways, which are important to carcinogenesis [20], contributed to PFAS signaling. This current work will expand into an in vivo rodent mammalian model to explore PFAS-dependent uterine weight changes (gravimetric changes) and gene expression changes including pathway enrichment.

## 2. Materials and Methods

### 2.1. Test Chemicals

All test chemicals in this study were dissolved or suspended in national ormulary (NF) grade corn oil (Spectrum Chemical, New Brunswick, NJ, USA, CAS 8001-30-7, lot#: 1IG1538, density: 0.9 g/mL, NF grade). Oral dosing solutions contained 0.5% polysorbate 20 (Tween 20) (Spectrum Chemical, CAS 9005-64-5, lot#: 2IK0104, density: 1.1 g/mL, NF grade) added to assist in dissolving or suspending chemicals in solution. Ethinyl Estradiol (EE2) (CAS 57-63-6, lot#: B20ZX01151, purity: 95%, USP grade) and β-estradiol 3-benzoate (EB) (CAS 50-50-0 lot#: BS197ZX09141, purity: 95%, USP grade) were purchased from BOC Sciences, Shirley, NY, USA. 4:2 fluorotelomer alcohol (4:2 FTOH) (CAS 2943-47-2, lot#: MKCK0293, density: 1.59 g/mL, purity: 97%), 6:2 fluorotelomer alcohol (6:2 FTOH) (CAS 642-42-7, lot#: MKCK4799, density: 1.652 g/mL, purity: 97%), Heptadecafluorooctanesulfonic acid-potassium salt (PFOS) (CAS 2795-39-3, lot#: BCBX5798, purity: 98%), and 2,2,3,3,4,4,5,5,6,6,7,7,8,8,9,9- Hexadecafluoro-1-10-decanediol (FC10-diol) (CAS 754-96-1, lot#: MKCR4342, purity: 97%) were purchased from Sigma, St. Louis, MO, USA. Perfluorooctanesulfonamide (PFOSA) (CAS 754-91-6, lot#: Q164-59, purity: 96%), Perfluorohexane sulfonate (PFHxS) (CAS 355-46-4, lot#:334800, purity: 95%), Nafion byproduct 2 (NBP2) (CAS 749836-20-2, lot#: 512400, purity: 97%), 8:2 fluorotelomer alcohol (8:2 FTOH) (CAS 678-39-7, lot#: 00015020, purity: 97%), 1H, 1H, 8H, 8H-Dodecafluoro-1,8-octanediol (FC8-diol) (CAS 90177-96-1, lot#: 00007478, purity: 98%), and Perfluorononanoic acid (PFNA) (CAS 375-95-1, lot#: 00010188, purity: 99%) were purchased from SynQuest Labs, Alachua, FL, USA. All purity information was determined by the chemical suppliers.

### 2.2. Animals

These studies were conducted on ovariectomized Sprague–Dawley (SD) rats obtained from Charles River Laboratories (Raleigh, NC). The rats were between 26–39 days of age at time of purchase. Once the rats arrived at US EPA (Research Triangle Park, NC, USA) they were held for one week before surgical clips were removed, after which they were held for an additional two weeks to allow for uterine regression and acclimation to facility. Rats were held in a controlled room in the animal handling facility, which was maintained at 20–22 °C and 45–55% relative humidity. The room also maintained a 12-h light/dark schedule. Rats were paired and placed in clear polycarbonate cages that contained laboratory grade, heat-treated pine shavings. The rats were provided with a diet of NTP 2000 (National Toxicology Program, NIEHS; Research Triangle Park, NC, USA) and 5 µm filtered municipal tap water, ad libitum. Studies were conducted with an approved Animal Care and Use Protocol (ACUP) from the US EPA’s Institutional Animal Care and Use Committee (IACUC) Protocol 23-01-002 that is accredited by the Association for Assessment and Accreditation of Laboratory Animal Care.

### 2.3. Uterotrophic Assay

The uterotrophic assay was conducted using the EPA’s Office of Chemical Safety and Pollution Prevention (OCSPP) Guideline 890.1600, which falls under the Endocrine Disruptor Screening Program [21]. To briefly describe the methods, rats were weight-ranked and randomly assigned into treatment groups using a simple randomization technique based on body weight and marked with a 5% picric acid solution. Rats were orally gavaged with one dose of test chemical daily for 4 consecutive days, with the exception of the EB test group, which was subcutaneously injected with 1 µg of EB in 0.1 mL of corn oil for 4 consecutive days. EB injection sites differed daily, using dorsal shoulder left lateral, dorsal shoulder right lateral, left ventral, and right ventral sites, respectively. Oral gavage doses were given at a volume of 2.5 mL/kg of body weight. Oral gavage of compounds of interest was performed at the same time each day. Doses were chosen based on previously published work in other labs [2,7]. Daily doses for each treatment were 250 mg/kg 4:2 FTOH, 250 mg/kg 6:2 FTOH, 125 mg/kg 8:2 FTOH, 10 mg/kg PFOSA, 50 mg/kg PFHxS, 10 mg/kg NBP2, 5 mg/kg PFNA, 2.5 mg.kg FC8-diol, 2 mg/kg FC10-diol, 5 mg/kg PFOS, and 0.1 mg/kg EE2.

Each experimental block contained EB and EE2 as the positive control, the control (corn oil), and between 4–5 test PFAS chemicals; and contained 3–6 rats per treatment. Seven hours after the final dose, rats were euthanized by decapitation while being restrained in a DecapiCone (Braintree Scientific, Braintree, MA, USA). Trunk blood was collected through a plastic funnel placed into a vacutainer tube (BD, Franklin Lakes, NJ, USA). Uterine tissue was resected whole, ensuring no ovarian remnants remained, and trimmed of extraneous tissue and fascia. Uterine tissue was weighed with luminal fluid intact to determine a wet weight. Tissue was then placed on absorbent paper, pierced multiple times with Dumont #3 tweezers, blotted of excess fluid, and reweighed for a blotted weight. Approximately 10–50 mg pieces of the right uterine horn were excised and placed in a clean 1.5 mL PCR tube (Eppendorf, Enfield, CT, USA), containing 1.0 mm zirconium oxide beads and 3.5 mm stainless steel UFO beads (Next Advance, Troy, NY, USA) and 250 µL of Tri-reagent (Sigma, St. Louis, MO, USA). Uterine tissue was homogenized by placing tubes in a Bullet Blender (Next Advance, Troy, NY, USA) tissue homogenizer for 10 min at 4 °C or until tissue was thoroughly homogenized. An additional 250 µL of Tri-reagent was placed into the tubes before storage in −80 °C freezer. The remainder of the uterine tissue was placed into a 10% formalin solution for future pathology analysis; uterine histopathology was not examined in this study. Trunk blood was allowed to clot for 30 min at room temperature, then tubes were centrifuged at 3000× *g* for 10 min to separate serum. Serum was placed into 1.5 mL PCR clean tubes and stored at −80 °C for future analysis.

### 2.4. Gene Expression

Homogenized uterine tissue was thawed, and RNA was extracted using the Tri Reagent protocol (Sigma, St. Louis, MO, USA). This protocol used chloroform and isopropanol to isolate RNA from DNA and proteins from the uterine tissue sample. RNA pellets were reconstituted in 10–20 µL of RNase-free water, depending on the amount of extracted RNA product. RNA samples were diluted 1:10 and examined on a NanoDrop Spectrophotometer (ThermoFisher, Waltham, MA, USA) for purity, aiming for a A260 to A280 ratio of 1.7 or higher. Approximately 6000 ng of RNA from each sample was then used in the GeneJET RNA Cleanup and Concentration Micro Kit (ThermoFisher, Waltham, MA, USA) using the supplied protocol paired with the DNase I treatment protocol, which was also supplied. The kit used spin columns to bind and wash each sample before the sample was eluted with 10–20 µL of RNase-free water. Samples were again examined, without dilution, on the NanoDrop Spectrophotometer, aiming for a A260 to A280 ratio between 2.0 and 2.1. Approximately 300 ng of concentrated RNA was then used in the RT^2^ First Strand Kit (Qiagen, Germantown, MD, USA) using the supplied protocol. This kit eliminated any remaining genomic DNA and synthesized cDNA for use in rt-qPCR applications. The cDNA sample was mixed with RNase-free water and RT^2^ SYBR Green Mastermix (Qiagen, Germantown, MD, USA) and pipetted onto a 96-well PCR plate at a volume of 25 µL per well. The plate used in this study was the RT^2^ Profiler PCR Array Rat Osmotic Stress (PARN-151Z, Qiagen, Germantown, MD, USA) containing 84 genes, including channel and transporter genes, oxidative stress genes, and hormone receptors, among others. Each array plate was run on the CFX Connect Real-Time PCR Detection System (Bio-Rad, Hercules, CA, USA) according to manufacturer protocol.

### 2.5. Pathway and Upstream Regulator Enrichment Analysis 

Pathway analysis of significant genes was conducted using ingenuity pathway analysis (IPA; QIAGEN, Redwood City, CA, USA). For each chemical tested, the data set was uploaded into IPA, and an expression analysis was performed on all experimental treatments. A comparison analysis was conducted across treatments to observe effects on biological networks and pathways. Pathway significance was determined using a z-score cutoff of >2 (activated) or <2 (inhibited) and *p*-value < 0.01.

### 2.6. Calculations and Statistical Analysis

All data analyses were performed using SAS 9.4 (Cary, NC, USA) and GraphPad Prism 8 (San Diego, CA, USA). Uterine weights (both wet and blotted) were measured and analyzed by analysis of variance (ANOVA), followed with comparison to control by Dunnett’s Multiple Comparison Test. Uterine tissue gene expression data were analyzed using the comparative cycle threshold (C_T_) methodology. The delta C_T_ values were calculated using the equation 2^−ΔΔC^_T_, which was then normalized to the mean C_T_ value of selected housekeeping genes. The housekeeping genes selected were *Hprt1* and *Rplp1*, as they were found to have non-significant ANOVA *p*-values (*p* > 0.01) by treatment and were shown to be resistant to changes induced by the estrogens. The delta C_T_ values were converted into fold induction values through the division of treated replicate delta C_T_ values by the mean delta C_T_ of control replicates for each gene examined. These fold induction values were log_10_ transformed prior to ANOVA. A false discovery rate adjustment was applied to ANOVA *p*-values using the two-stage linear step-up procedure of Benjamini, Krieger, and Yekutieli at a rate of 5%.

## 3. Results

### 3.1. Uterotrophic Assays, Ovariectomized Adult Rats

**Figure 1:**
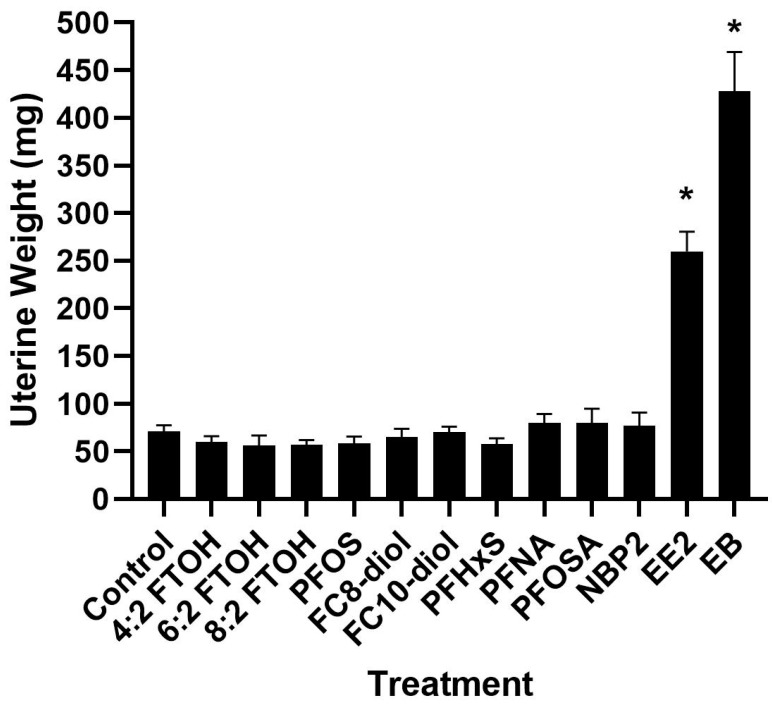
Uterine wet weight. Uterotrophic assay in adult ovariectomized rats. Wet uterine wet following exposure to ethinyl estradiol (EE2), estradiol benzoate (EB), 6:2 Fluorotelomere alcohol (6:2FTOH), 8:2 FTOH, 4:2 FTOH, Perfluorooctane sulfonic acid (PFOS), 1H,1H,8H,8H-Perfluorooctane-1,8-diol (FC8-diol) and 1H,1H,10H,10H-Perfluorodecane-1,10-diol (FC10-diol), Perfluorohexane sulfonate (PFHxS), Perfluorononanoic acid (PFNA), and Perfluorooctanesulfonamide (PFOSA), nafion byproduct 2 (NB2). * Significant treatment effect shown via ANOVA (*p* = 0.05) with comparison to the control by Dunnett’s Multiple Comparison Test. No significant treatment effects were found after PFAS exposure.

**Figure 2:**
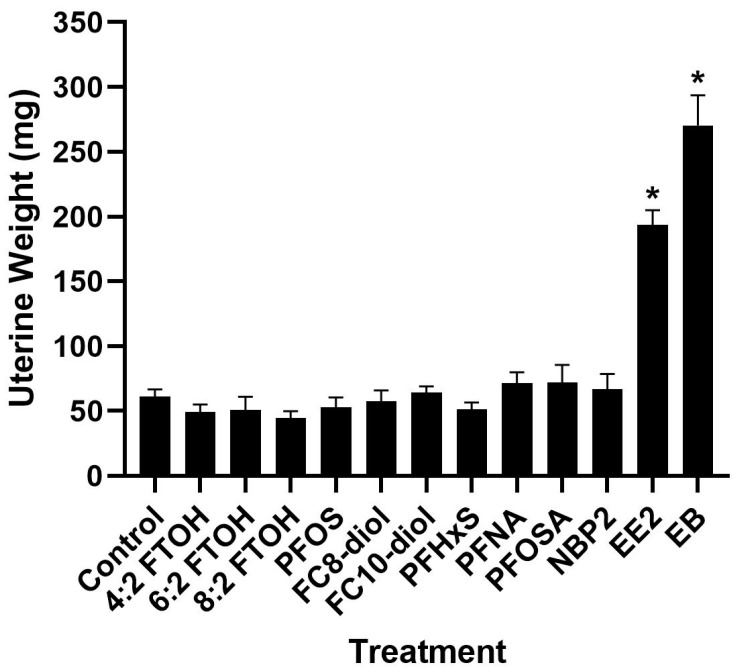
Uterine blotted (dry) weight. Uterotrophic assay in adult ovariectomized rats. Blotted following exposure to ethinyl estradiol (EE2), estradiol benzoate (EB), 6:2 Fluorotelomer alcohol (6:2FTOH), 8:2 FTOH, 4:2 FTOH, Perfluorooctane sulfonic acid (PFOS), 1H,1H,8H,8H-Perfluorooctane-1,8-diol (FC8-diol) and 1H,1H,10H,10H-Perfluorodecane-1,10-diol (FC10-diol), Perfluorohexane sulfonate (PFHxS), Perfluorononanoic acid (PFNA), and Perfluorooctanesulfonamide (PFOSA), nafion byproduct 2 (NB2). * Significant treatment effect found via ANOVA (*p* = 0.05) with comparison to the control by Dunnett’s Multiple Comparison Test. No significant treatment effects were found after PFAS exposure.

The potential in vivo estrogenicity of a panel of PFAS was evaluated in the rat ovariectomized uterotrophic assay. Data from the 5-day uterotrophic assay are reported in Figure 1 and Figure 2 and Table 1; Table 1 includes the data that are graphically represented in Figure 1 and Figure 2. The data in Figure 1 and Figure 2 and Table 1 are generated from uterine weight or animal body weight measurements. In this study, none of the PFAS screened produced statistically significant changes in uterine weight (wet or blotted) compared to vehicle controls. The positive control compounds of subcutaneous estradiol benzoate and ethinyl significantly increased uterine weight (Figure 1 and Table 1). Analysis of relative uterine weight (data not shown) also only produced significant changes with estrogen exposure.

### 3.2. Osmotic Gene Arrays

**Figure 3:**
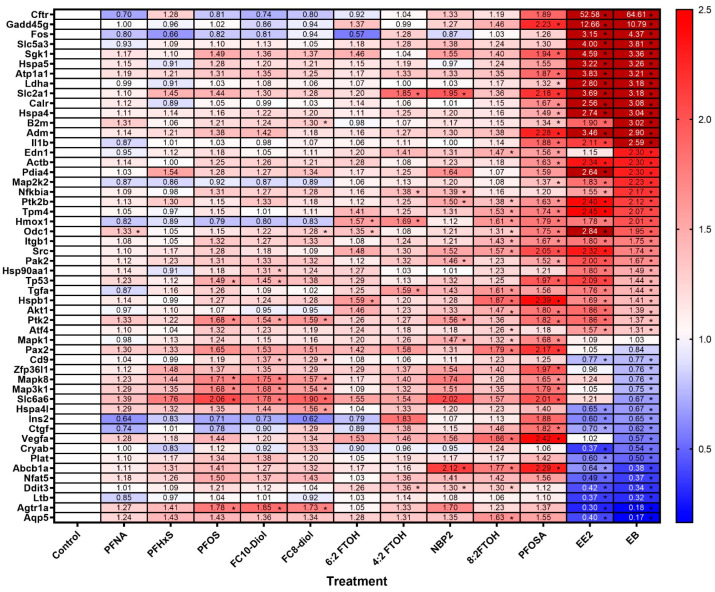
Heatmap of PFAS-dependent changes in uterine mRNA gene expression using osmotic stress RT-qPCR gene array. Genes with statistically significant changes found via ANOVA have a value of *p* < 0.01(*). Fold change showing up- or downregulation of significant osmotic stress target genes from RT-qPCR arrays. A false discovery rate was applied using the two-stage step-up method of Benjamini, Krieger, and Yekutieli at a rate of 5%. Cells denoted in blue are decreased fold change and those in red are elevated fold change values above 2.5. An asterisk denotes significant fold change values.

**Figure 4:**
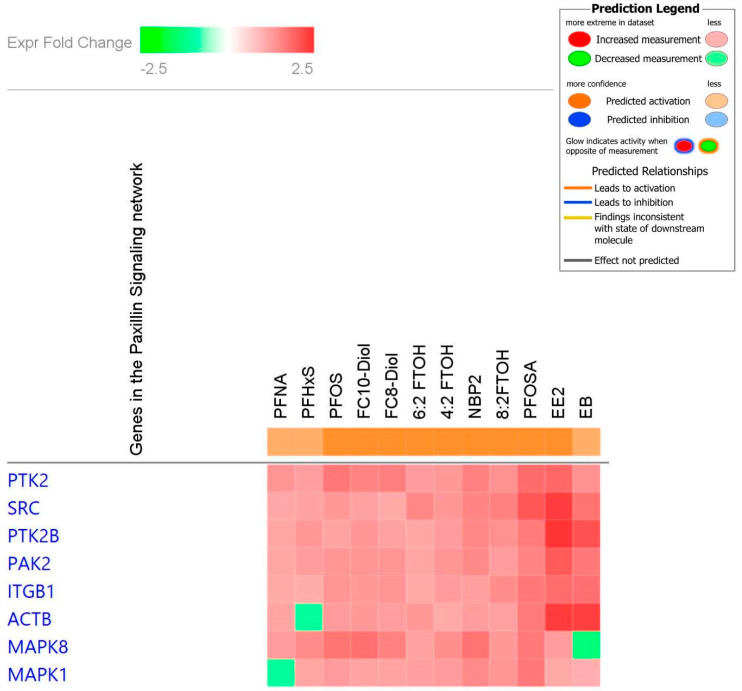
Ingenuity pathway analysis of PFAS-dependent changes in uterine mRNA gene expression (*n* = 5–6 animals per PFAS) using data from the uterine osmotic gene array as well as positive controls ethinyl estradiol and estradiol benzoate. Genes within the paxillin signaling network are denoted by color change. Z-score and adjusted *p* value filters were set to ≥2.5 and ≤0.05, respectively.

**Figure 5:**
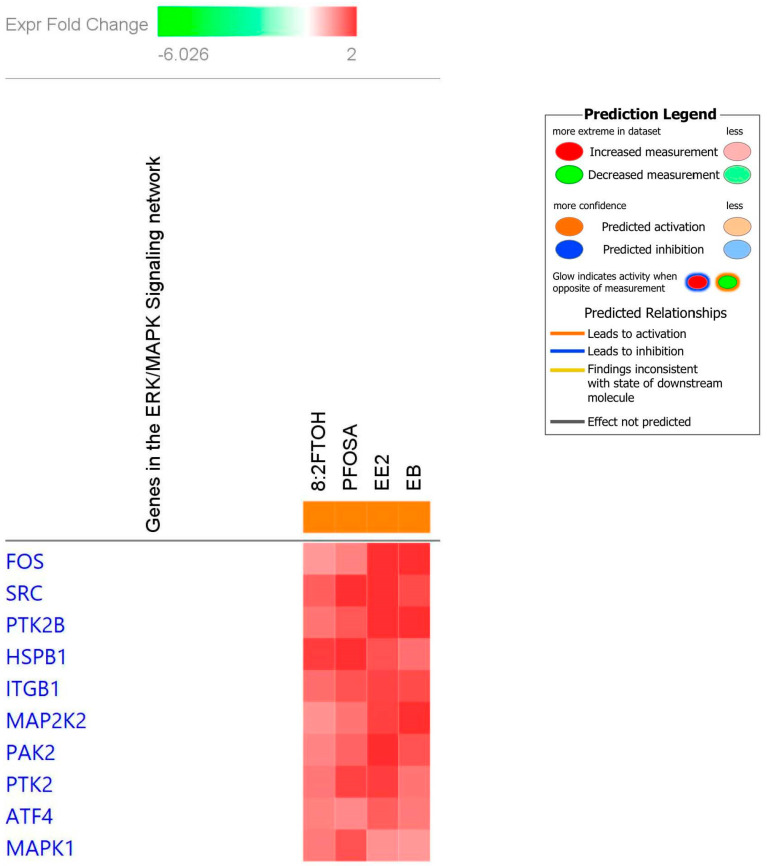
Ingenuity pathway analysis of data from the uterine osmotic gene array showing PFAS (PFOSA and 8:2 FTOH)-dependent changes in gene activation on the MAPK pathway. PFOSA and 8:2 FTOH were the two compounds most like the estrogens in their activation pattern. IPA revealed these two PFAS most strongly activated the MAPK signaling pathway. The genes enriched in the MAPK network are denoted below with their degree of activation.

To increase the sensitivity of the uterotrophic assay beyond the gravimetric uterine changes, molecular osmotic changes were quantified in extracted uterine mRNA with an osmotic stress RT-qPCR gene expression array. The osmotic array includes 84 genes which are key to changes in osmolarity including transporters, cytoskeleton, oxidative stress, cell cycle control, apoptosis, hormone, receptors, and transcription/translation arrest genes and can provide mechanistic support to weight changes that can be observed in the uterotrophic assay. Data from the uterine osmotic gene array were used in (1) a heatmap as shown in Figure 3 and (2) with ingenuity pathway analysis as shown in Figure 4 and Figure 5. Uterine gene expression array analysis with an osmotic stress array yielded multiple positive findings as demonstrated in the heatmap (Figure 3). The positive control estrogens (EE2 and EB) induced significant changes in the osmotic genes, with the majority of the changes showing an upregulation of expression. Analysis with the array revealed that PFOSA and 8:2 FTOH were the treatments that displayed the most significant gene fold changes. PFOSA and 8:2 FTOH induced upregulation of multiple osmotic gene changes similar in magnitude to the positive control estrogens, as seen in Figure 2. *Slc6a6* was upregulated across all of the PFAS, though not all at a level of significance, trending similar to EE2 but opposite in direction from EB. PFNA had the most genes trending downward. Multiple genes (*Fos*, *Cftr*, *Map2k2*, *Hmox1*, and *Ins2*) demonstrated downregulation trends across five PFAS (PFNA, PFHxS, PFOS, FC10-diol and FC8-diol). Similar to the estrogens, several PFAS (PFNA, PFHxS, PFOS, FC10-diol, 6:2FTOH) induced downregulation of *Ins2*, the gene encoding for insulin. The PFAS upregulated a group of genes (*Cd9*, *Hspa4l*, *Vegfa*, *Cryab*, *Plat*, *Abcb1a*, *Nfat5*, *Ddit3*, *Ltb*, *Agtr1a*, and *Aqp5*) that were downregulated by both estrogens.

In addition to the individual level gene changes, ingenuity pathway analysis demonstrated an enrichment of the paxillin gene pathway when surveyed across all the PFAS tested, including the genes *Ptk2*, *Src*, *Ptk2b*, *Pak2*, *Itgb1*, *Actb*, *Mapk8*, and *Mapk1* (Figure 4). ingenuity pathway analysis across PFOSA and 8:2FTOH, the PFAS with genetic activation most similar to estrogens, showed that these two chemicals activated the *Mapk* pathway with enrichment of the following genes *Fos*, *Src*, *Ptk2b*, *Hspb1*, *Itgb1*, *Map2k2*, *Pak2*, *Atf4*, and *Mapk1* (Figure 5).

## 4. Discussion

The findings here report the results of the ovariectomized uterotrophic assay in adult rats that were orally exposed to a panel of PFAS at single-dose levels. The PFAS that were screened in the current assay included PFNA, PFHxS, PFOS, FC10-diol, FC8-diol, 8:2 FTOH, 6:2 FTOH, 4:2 FTOH, Nafion Byproduct2, and PFOSA. The PFAS used in this study were chosen based on previous estrogenic or hormonal activity in vitro [2], with hormonal sensitive endpoints in fish (activation of vitellogenic or estrogen-responsive genes a) [7,8] or with changes in puberty onset [5]. Previously published work with low doses of PFOA demonstrated increased wet uterine wet at only one dose with the immature rat uterotrophic assay [22], a different version of the uterotrophic assay which screens chemicals in young pre-pubertal animals. This study used the adult ovariectomized uterotrophic assay. Even though chemicals have been shown to induce estrogen-sensitive pathways in fish or with in vitro or receptor-mediated assays, the PFAS screened here did not induce significant uterine weight or fluid changes in the adult ovariectomized uterotrophic assay. The uterotrophic assay was developed as part of the standard guideline testing at the EPA under the Endocrine Disruptor Screening Program (OCSPP Guideline 890.1600). These findings can be useful for the validation and assessment of endocrine disrupting screenings and methods spanning in vitro to in vivo extrapolation [23].

To build mechanistic understanding of PFAS-dependent changes in the uterus, osmotic gene arrays were used and compared to the estrogens. Osmotic array data were used to generate a heatmap and were also processed through ingenuity pathway analysis to show what pathways might be involved in PFAS signaling. Estrogens induce a significant increase in uterine weight, serving as the positive controls in the uterotrophic assays, with many of the changes thought to relate to osmotic changes. Individual gene expression upregulation was enriched across the array with PFAS treatment. The PFAS screened in the osmotic array tended to induce upregulation of osmotic genes, but a few PFAS did contribute to some osmotic gene downregulation. Across five PFAS (PFNA, PFHxS, PFOS, FC10-diol, and FC8-diol), trends of enrichment in downregulation were visible (*Fos*, *Cftr*, *Map2k2*, *Hmox1*, and *Ins2*) (Figure 3). Similar to the estrogens, several PFAS (PFNA, PFHxS, PFOS, FC10-diol, 6:2FTOH) induced down regulation of *Ins2*, the gene encoding insulin. *Ins2* is important for glucose tolerance, and *Ins* null mice have pups that experience intrauterine growth restriction in utero [24]. PFAS exposure in laboratory animal studies and human cohorts is associated with small decreases in birth weight [25,26], and decreased birth weight is listed by the CDC/ATSDR on its document on the health effects of PFAS [27](ATSDR 2023). Also, multiple studies of associations of PFAS with diabetes have been conducted with mixed results [28,29,30].

To understand broadly what pathways or groups of genes are activated across biological systems, we analyzed the osmotic array data with ingenuity pathway analysis. In the current study, PFAS-dependent changes in uterine gene activation using ingenuity Pathway analysis found an enrichment of genes involved in the paxillin pathway across all PFAS screened with similar gene activation seen with estrogen exposure. The PFAS-dependent enrichment upregulated the family of genes contributing to the paxillin family signaling include *Ptk2*, *Src*, *Ptk2b*, *Pak2*, *Itgb1*, *Actb*, *Mapk8*, and *Mapk1* (Figure 4). Mechanistically, paxillin serves as a substrate for SHP-2 (Src homology-2 domain-containing protein tyrosine phosphatase-2), which is involved in tumor invasion and metastasis, apoptosis in cancer, tumor cell proliferation and cell cycle changes, DNA damage and replication in cancer, and drug resistance in cancer [31]. SHP-2 is involved in signaling cascades that are implicated in cancer (Noonan syndrome, LEOPARD syndrome, and childhood leukemia) [31]. In vitro studies with PFOA exposure in a uterine cell line showed that PFOA treatment promoted invasion and migration of cancer cells mediated through activation of ERK/mTOR signaling [32], which cross talks to the MAPK pathway, another potential upstream mediator of paxillin. In our study, ingenuity pathway analysis showed PFOSA and 8:2 FTOH activated MAPK pathways; among the PFAS screened in this study, PFOSA and 8:2 FTOH were most similar to the estrogens in their genetic enrichment profile in the osmotic array. Similar to the current study, [20] also demonstrated involvement of paxillin signaling in PFAS dependent changes in liver cancer cells. In this previously published study, PFAS (PFOA, Perfluorooctadecanoic acid (PFODA)) were shown to bind and inhibit the tumor suppressor gene SHP-2, an upstream modulator to paxillin, resulting in increased levels of paxillin in HEPG2 liver cells [20]. In [20], the potency of the paxillin pathway activation was dependent on PFAS chain length with the longer PFAS serving as more potent activators of the pathway. The SLC6 genes encode transporters that are found in tissues throughout the nervous system, gut, and kidney [33]. In previous publications, SLC6A6 expression has been shown to drive tumorigenesis and affects clinical outcomes in cancer [34,35]. In this study, SLC6A6 was upregulated across all the PFAS in a pattern similar to EE2.

Biological insight into what pathways may be important for signaling was provided through ingenuity pathway analysis, which revealed enrichment of the MAPK pathway with the two most estrogen-like PFAS (PFOSA and 8:2 FTOH) and enrichment of the paxillin pathway with all of the PFAS screened. Individual genes were also impacted by PFAS exposure with global downregulation of the insulin gene *Ins2* across PFAS. Despite non-significant weight or gravimetric changes on the uterus with PFAS exposure, there were significant upregulation of osmotic pathways with PFAS exposure and a lesser degree of downregulation of osmotic pathways. These finding provide an understanding of signaling in the PFAS-exposed uterus that can inform adverse outcome pathways and provide potential biomarkers for future work.

## Figures and Tables

**Table 1:** Uterotrophic assay gravimetric endpoints including body weight gain, uterine wet weight, uterine blotted weight, and uterine fluid (mean ± standard error) in ovariectomized females exposed to various PFAS chemical treatments. Asterisks indicate *p*-value < 0.01.

**Treatment**	**Control**	**EB**		**EE2**		**4:2 FTOH**		**6:2 FTOH**		**8:2 FTOH**		**PFOSA**
	0 mg/kg	1 µg i.p.		0.1 mg/kg		250 mg/kg		250 mg/kg		125 mg/kg		10 mg/kg
	*n* = 18	*n* = 17		*n* = 17		*n* = 8		*n* = 5		*n* = 7		*n* = 6
Initial Body Weight (g)	323.9 ± 11.6	329.6 ± 9.3		329.2 ± 8.3		321.8 ± 9.4		312.0 ± 15.8		316.9 ± 16.6		310.5 ± 8.1
Final Body Weight (g)	333.7 ± 9.5	323.8 ± 9.2		319.0 ± 7.2		324.4 ± 9.1		326.3 ± 17.3		329.5 ± 17.6		314.5 ± 10.7
Body Weight Gain (g)	14.2 ± 3.6	2.7 ± 1.4		4.9 ± 1.9		10.6 ± 1.8		14.0 ± 2.0		15.8 ± 1.5		6.3 ± 3.3
Uterine Wet Weight (mg)	71.2 ± 6.0	428.0 ± 41.1 *		259.7 ± 20.7 *		59.7 ± 6.0		55.8 ± 11.0		57.0 ± 5.1		79.5 ± 15.3
Uterine Blotted Weight (mg)	61.2 ± 5.3	270.3 ± 23.2 *		193.4 ± 11.4 *		49.3 ± 5.7		50.5 ± 10.3		44.6 ± 5.1		71.9 ± 13.7
Uterine Fluid (mg)	10.0 ± 1.2	157.7 ± 26.4 *		66.3 ± 11.0 *		10.4 ± 2.4		5.3 ± 1.2		12.3 ± 1.4		7.7 ± 1.7
							
**Treatment**	**NBP2**		**PFHxS**		**PFNA**		**FC8-diol**		**FC10-diol**		**PFOS**	
	10 mg/kg		50 mg/kg		5 mg/kg		2.5 mg/kg		2 mg/kg		5 mg/kg	
	*n* = 7		*n* = 7		*n* = 6		*n* = 6		*n* = 5		*n* = 5	
Initial Body Weight (g)	323.0 ± 11.1		335.3 ± 14.2		350.0 ± 6.9		358.8 ± 14.8		365.6 ± 11.3		351.0 ± 11.0	
Final Body Weight (g)	333.2 ± 8.9		332.2 ± 21.8		349.9 ± 8.3		361.5 ± 15.6		369.7 ± 9.1		352.3 ± 10.2	
Body Weight Gain (g)	7.5 ± 0.8		9.3 ± 2.4		7.1 ± 2.6		8.8 ± 1.3		8.6 ± 2.0		12.2 ± 3.7	
Uterine Wet Weight (mg)	76.9 ± 13.9		57.9 ± 5.8		79.8 ± 9.3		64.9 ± 8.7		70.5 ± 5.4		58.1 ± 7.3	
Uterine Blotted Weight (mg)	66.7 ± 11.8		51.3 ± 5.2		71.6 ± 8.2		57.5 ± 8.2		63.9 ± 4.9		52.8 ± 7.6	
Uterine Fluid (mg)	10.3 ± 2.3		6.5 ± 0.6		8.2 ± 1.4		7.4 ± 1.1		6.6 ± 0.8		5.3 ± 0.9	

## Data Availability

Data will be made available upon request.
